# Evolution of aromatic amino acid metabolism in plants: a key driving force behind plant chemical diversity in aromatic natural products

**DOI:** 10.1098/rstb.2023.0352

**Published:** 2024-11-18

**Authors:** Ryo Yokoyama

**Affiliations:** ^1^ Max Planck Institute of Molecular Plant Physiology, Potsdam, Am Mühlenberg 1 14476, Germany

**Keywords:** aromatic amino acid, aromatic natural products, enzyme evolution, negative feedback inhibition

## Abstract

A diverse array of plant aromatic compounds contributes to the tremendous chemical diversity in the plant kingdom that cannot be seen in microbes or animals. Such chemodiversity of aromatic natural products has emerged, occasionally in a lineage-specific manner, to adopt to challenging environmental niches, as various aromatic specialized metabolites play indispensable roles in plant development and stress responses (e.g. lignin, phytohormones, pigments and defence compounds). These aromatic natural products are synthesized from aromatic amino acids (AAAs), l-tyrosine, l-phenylalanine and l-tryptophan. While amino acid metabolism is generally assumed to be conserved between animals, microbes and plants, recent phylogenomic, biochemical and metabolomic studies have revealed the diversity of the AAA metabolism that supports efficient carbon allocation to downstream biosynthetic pathways of AAA-derived metabolites in plants. This review showcases the intra- and inter-kingdom diversification and origin of committed enzymes involved in plant AAA biosynthesis and catabolism and their potential application as genetic tools for plant metabolic engineering. I also discuss evolutionary trends in the diversification of plant AAA metabolism that expands the chemical diversity of AAA-derived aromatic natural products in plants.

This article is part of the theme issue ‘The evolution of plant metabolism’.

## 1. Introduction

Plants are able to synthesize tremendously diverse natural products, displaying the remarkable diversity of phytochemicals with a wide range of chemical structures and biological activities. It is widely accepted that such chemical diversity in the plant kingdom has emerged as an evolutionary consequence of the adaptation to challenging environmental conditions on land [1–3]. Among these compounds, aromatic natural products stand out for their remarkable structural diversity and functional versatility. Because of the unique physical and chemical properties of the aromatic ring(s) in their chemical structure, such as UV-absorbance, hydrophobicity and reactivity, aromatic natural products play critical roles in various aspects of plant biology, such as pigmentation, defence against pathogens, pollinator attraction, regulation of growth and development and adaptation to environmental stresses. Some of these plant-derived aromatic compounds have been used by human beings as nutraceuticals, pharmaceuticals and biomaterials [4]. Owing to the paramount and broad importance of these aromatic plant natural products in both plants and humans, the biosynthesis of aromatic natural products has been a promising target of metabolic engineering, aiming to increase their accumulation in plants. Unlike microbial chemical production, which requires an external carbohydrate supply, plants are anticipated to be a more sustainable platform that produces high-value aromatics than microbes, as plants can use CO_2_ as a sole carbon source for chemical production [5–8]. For example, the production and accumulation of suberin, an aromatic polymer with its chemically recalcitrant nature, have been intensively investigated for plant-based carbon capturing and sequestration [9]. However, plant chemical engineering often faces several challenges, including slow growth, limited capacity for genetic modification and too tight integration between plant growth and primary metabolism. To overcome them, an understanding of plant metabolism evolution—how plants have evolved to produce large amounts of diverse natural products—may help us harness their natural metabolic innovations for sustainable plant-based chemical production [8].

**Figure 1.**
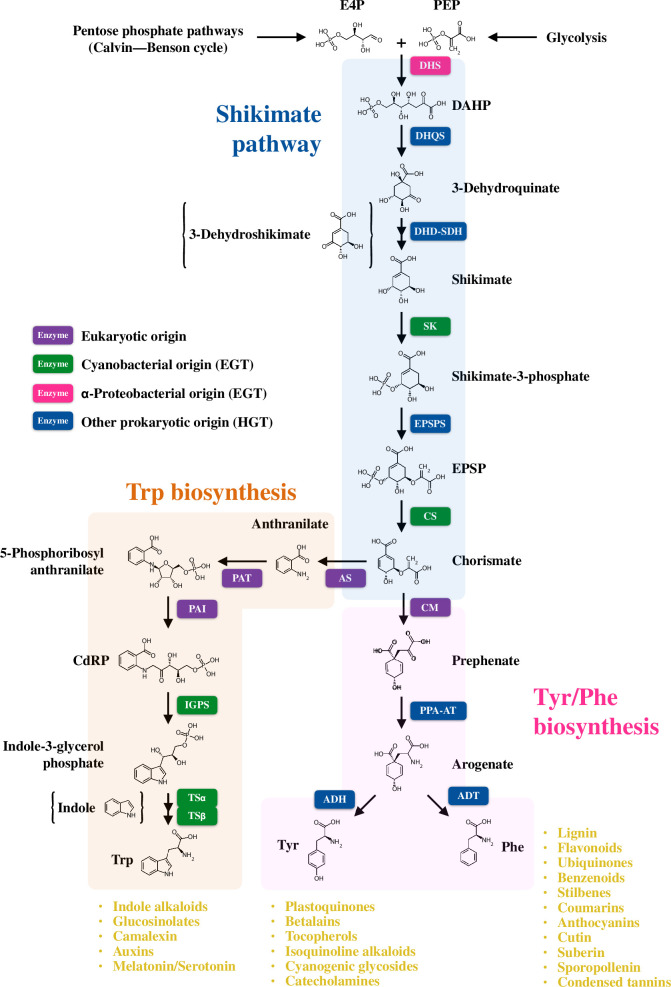
A pathway map and origin of the shikimate and aromatic amino acid biosynthetic pathways in plants. In plants, the shikimate pathway leads to the biosynthesis of AAAs, which are not only required for protein synthesis but also used as precursors of numerous AAA-derived aromatic natural products (yellow letters). The intermediates of the DHD-SDH and TS enzyme-catalysed reactions (3-dehydroshikimate and indole, respectively) are shown in brackets. Enzymes in the shikimate and AAA biosynthetic pathways are of mosaic origin, highlighted by purple (eukaryotic origin), green (cyanobacterial origin), magenta (α-proteobacterial origin) and blue (other prokaryotic origin). DHS, 3-deoxy-d-*arabino*-heptulosonate 7-phosphate (DAHP) synthase; DHQS, 3-dehydroquinate synthase; DHD-SDH, 3-dehydroquinate dehydratase-shikimate dehydrogenase; SK, shikimate kinase; EPSPS, 5-*enol*pyruvylshikimate 3-phosphate (EPSP) synthase; CS, chorismate synthase; AS, anthranilate synthase; PAT, phosphoribosylanthranilate transferase; PAI, phosphoribosylanthranilate isomerase; IGPS, indole-3-glycerol phosphate synthase; TSα, tryptophan synthase α subunit; TSβ, tryptophan synthase β subunit; CM, chorismate mutase; ADT, arogenate dehydratase; ADH, arogenate dehydrogenase; PPA-AT, prephenate aminotransferase; E4P, d-erythrose 4-phosphate; PEP, phospho*enol*pyruvate and CdRP, 1-(*o*-carboxyphenylamino)-1-deoxy-ribulose 5-phosphate; EGT, endosymbiotic gene transfer; HGT, horizontal gene transfer.

Aromatic amino acids (AAAs)— l-tyrosine, l-phenylalanine and l-tryptophan (Tyr, Phe and Trp, respectively) are not only three of the 20 proteogenic amino acids but also serve as precursors for many aromatic specialized metabolites in plants. Because of the lack of AAA biosynthetic capability, animals obtain AAAs through their diet [10]. In microbes and plants, AAA biosynthesis is initiated in the shikimate pathway, into which an abundance of carbon precursors are directed from central carbon metabolic pathways—pentose phosphate pathway (Calvin–Benson cycle in photosynthetic organisms) and glycolysis. The shikimate pathway is composed of seven enzymatic reactions to generate chorismate at the final step, where the pathway is branched into Tyr/Phe and Trp biosynthesis, respectively (figure 1). Unlike cytoplasmic localization of the shikimate pathway in microbes, plant shikimate and AAA biosynthetic pathways are mainly localized in plastids [4,11]. Besides using AAAs for protein synthesis, AAAs are further metabolized into AAA-derived aromatic natural products at the interface of primary and specialized metabolic pathways [4,12,13]. Tyr is converted into various pigment chemicals, including plastoquinones for electron careers, betalains for red pigments and tocopherols (vitamin E) for antioxidants [14–16]. Myriads of Phe-derived natural products have played a crucial role in the aquatic-to-terrestrial colonization and subsequent thriving of land plants to cope with various harsh environmental stresses on land, such as gravity and UV. In particular, land plants have developed the complex biosynthetic pathway of phenylpropanoids, the largest class of plant natural products, next to terpenoids, that include flavonoids, anthocyanin pigments, tannins and the principal cell wall component lignin [11,17–20]. Trp plays a crucial as precursors of bioactive compounds, including phytohormone auxins, plant defence compounds indole glucosinolates and camalexin and stress management phytochemicals serotonin and melatonin as well as pharmatucials, such as cancer drugs glucobrassicin and vincristine produced in Brassicaceae and *Catharanthus roseus* [21–25]. Major plant aromatic natural products are shown in figure 1.

Unlike the highly diversified specialized metabolism of AAA-derived natural products in plants, primary metabolism, including amino acid metabolism, is assumed to be conserved across the kingdoms. Whereas AAA metabolism mostly shares common enzymes across life forms, the regulation, location and integration with other metabolic pathways in plants have evolved distinctly owing to their sessile nature. In most cases, this diversification has emerged to support the high capacity of the downstream specialized metabolism via the optimization of their precursor availability. This trend highlights the importance of fine-tuning AAA metabolism in the diversification of plant aromatic natural products. Among the more than 20 enzymes involved in AAA biosynthesis and catabolism, I focus in particular on several enzymes that mediate key steps in the regulation of AAA metabolism and display remarkable diversification of their enzyme functions (e.g. negative feedback inhibition). This review aims to highlight general trends in the diversification of AAA metabolism and its evolutionary role in the remarkable chemical diversity of aromatic natural products in the plant kingdom.

## 2. Mosaic origin of aromatic amino acid biosynthesis in plants

The genomes of plants (Archaeplastida) have at least three origins. Through endosymbiosis with an α-proteobacterial ancestor, a eukaryotic host gained mitochondria. This was followed by subsequent endosymbiosis with a cyanobacterial ancestor, which resulted in the formation of plastids [26]. Since all three lineages possess their primary metabolic pathways, ancestral plants have acquired multiple metabolic genes from different origins via horizontal gene transfer (HGT) from the three major origins through an endosymbiotic event (endosymbiotic gene transfer, EGT) or occasionally from the other lineages and selectively lost some of them during plant evolution. As a result, genes and enzymes in plant primary metabolism have different origins [1]. Such mosaic origin can be seen in various plant primary metabolic pathways, including the Calvin–Benson cycle, plastidic 2-C-methyl-d-erythritol 4-phosphate pathway and galactolipid biosynthetic pathway [1,27–30]. Similarly, many of the enzymes in the plant shikimate and AAA biosynthetic pathways are, although mainly localized in plastids, not fully derived from a cyanobacterial ancestor but of mosaic origin [31]. For example, plant 3-deoxy-d-*arabino*-heptulosonate 7-phosphate synthase (DAHP synthase or DHS) enzymes belong to the type II group that includes DHS enzymes of several bacteria (e.g. *Mycobacterium tuberculosis* and some α-proteobacteria) but is phylogenetically distinct from the type I group containing *Escherichia coli*, yeast and cyanobacteria DHS enzymes. This phylogenetic information indicates that plant DHS genes are derived from bacteria having type II DHS genes, rather than EGT from cyanobacteria [31,32]. Plant shikimate kinase and chorismate synthase enzymes are evolutionarily close to cyanobacterial orthologs, while plant chorismate mutase (CM) is probably of eukaryotic origin [31,33]. The plant prephenate aminotransferase (PPA-AT) gene was probably transferred from a Chlorobi/Bacteroidetes ancestor [34]. The other enzymes in the shikimate and Tyr/Phe pathways, such as 3-dehydroquinate synthase, 5-*enol*pyruvylshikimate 3-phosphate synthase, and arogenate dehydratase/dehydrogenase (ADT/ADH), are probably obtained via HGT from other prokaryotes rather than EGT. In Trp biosynthesis, the first three enzymes, anthranilate synthase (AS), phosphoribosylanthranilate transferase, and phosphoribosylanthranilate isomerase, originate from the eukaryotic nucleus, whereas the last three enzymes, indole−3-glycerol phosphate synthase and the α and β subunits of tryptophan synthase (TSα and TSβ, respectively) were acquired through cyanobacterial EGT [35]. Collectively, the plant plastidic AAA biosynthetic pathway is of mosaic origin, with many enzymes replaced by those with noncyanobacterial origin [1], as summarized in figure 1. However, it remains unclear how ancestral plants selected enzymes of specific origin for each reaction. As discussed later, plastids (chloroplasts) are an ideal location to obtain carbon and energy resources sufficient to support the energy-intensive AAA biosynthetic pathway. Evolutionarily selected enzymes might be the most biochemically suitable or easy-to-fine-tuned to maximize their enzymatic activity in plastids.

## 3. 3-deoxy-d-*arabino*-heptulosonate 7-phosphate synthase

**Figure 2.**
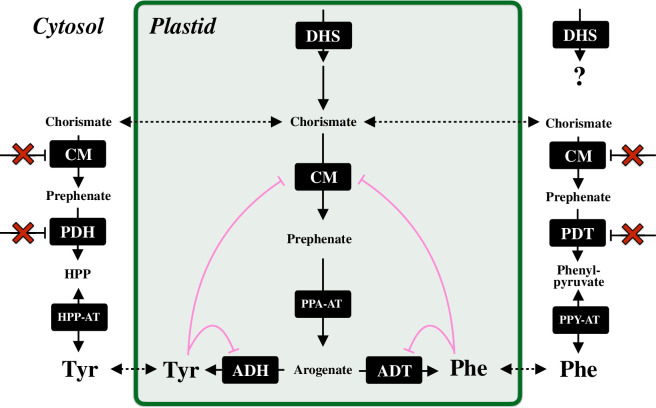
A model of plastidic and cytosolic post-chorismate pathways. Chorismate is exported from plastids through an unknown transporter and is branched to cytosolic Tyr and Phe biosynthetic pathways (left and right, respectively). A deregulated CM isoform converts chorismite into prephenate in the cytosol. Prephenate is subsequently dehydrogenased and dehydratased into HPP and phenylpyruvate by PDH and PDT enzymes that are insensitive to Tyr and Phe, respectively. HPP and phenylpyruvate are reversibly transaminated into Tyr and Phe, respectively. The contribution of cytosolic DHS enzymes to AAA metabolism in the cytosol remains unclear. DHS, 3-deoxy-D-*arabino*-heptulosonate 7-phosphate synthase; CM, chorismate mutase; PDT, prephenate dehydratase; ADT, arogenate dehydratase; PDH, prephenate dehydrogenase; ADH, arogenate dehydrogenase; PPA-AT, prephenate aminotransferase; PPY-AT, phenylpyruvate aminotransferase; HPP-AT, 4-hydroxylphenylpyruvate aminotransferase; PAH, phenylalanine hydroxylase and HPP, 4-hydroxylphenylpyruvate.

Given that the shikimate pathway connects the upstream carbon metabolism with the biosynthesis of AAAs and AAA-derived natural products, the carbon flux into the shikimate pathway must be strictly yet flexibly regulated at its entry reaction in response to the upstream carbon availability and the metabolic demand from the downstream pathways. DHS enzymes catalyse the first committed reaction of the shikimate pathway that integrates phospho*enol*pyruvate (PEP) and d-erythrose−4-phosphate (E4P), derived from glycolysis and the Calvin–Benson cycle, respectively (figure 1; [4,32]). DHS enzymes are mainly localized in the plastids (chloroplasts), while several studies have proposed cytosolic DHS enzymes that may contribute to chorismate production in the cytosol (figure 2; [36–38]). Two distinct DHS activities that require manganese and cobalt have been isolated from plastid and cytosolic fractions, respectively [32,39,40]. However, the physiological role and the identities of cytosolic DHS activity remain undescribed.

**Figure 3.**
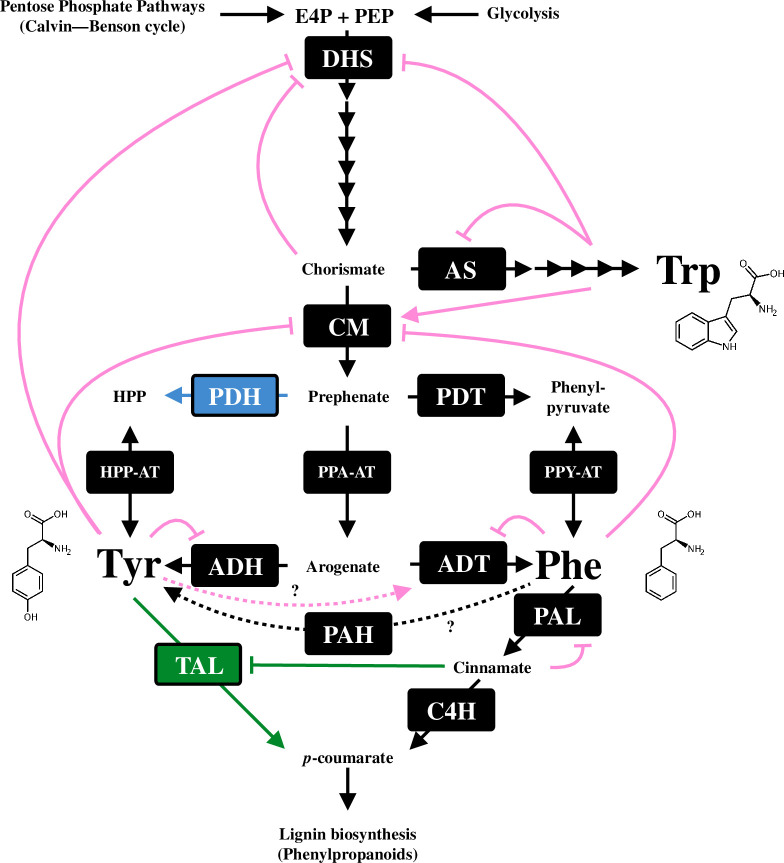
Feedback regulatory network in aromatic amino acid biosynthesis in plants. The pink lines with an arrowhead or a hash indicate known feedback activation or inhibition, respectively, while the dashed lines denote poorly characterized reactions or regulations that require further investigation. Blue or green colours indicate the reaction, enzyme and regulation that are known to be legume- or grass-specific, respectively. DHS, 3-deoxy-d-*arabino*-heptulosonate 7-phosphate synthase; AS, anthranilate synthase; CM, chorismate mutase; PDT, prephenate dehydratase; ADT, arogenate dehydratase; PDH, prephenate dehydrogenase; ADH, arogenate dehydrogenase; PPA-AT, prephenate aminotransferase; PPY-AT, phenylpyruvate aminotransferase; HPP-AT, 4-hydroxylphenylpyruvate aminotransferase; PAH, phenylalanine hydroxylase; PAL, phenylalanine ammonia-lyase; TAL, tyrosine ammonia-lyase; C4H, cinnamate 4-hydroxylase; E4P, d-erythrose 4-phosphate; PEP, phospho*enol*pyruvate and HPP, 4-hydroxylphenylpyruvate.

In microbes, which generally do not require extensive production of aromatic natural products, DHS enzymes are solely regulated by feedback inhibition by either AAA [13,41]. By contrast, plant DHS enzymes are subjected to a more complex negative feedback inhibition network that is mediated by various compounds including Tyr, Trp, chorismate and caffeate, as well as Tyr-derived and Trp-derived compounds such as homogentisate and indole-3 pyruvate, respectively ([42,43]; figure 3). With multiple isoforms exhibiting distinct sensitivities to these effector molecules, plants selectively express specific DHS isoforms to modulate the biosynthetic activities of AAAs and AAA-derived metabolites. In *Arabidopsis thaliana*, for instance, the DHS isoform susceptible to inhibition by Tyr or Trp is predominantly expressed in young tissues where protein synthesis actively takes place for growth. This expression pattern probably facilitates the monitoring of amino acid concentrations required for protein synthesis in still-developing young tissues. On the other hand, other DHS isoforms with insensitivity to Tyr or Trp are more abundantly expressed in mature tissues where AAA-derived metabolite synthesis takes precedence over protein synthesis [43]. A recent suppressor screening of Arabidopsis Tyr-deficient *tyra2* mutant identified several point mutations (*suppressor of tyra2*, *sota*) on the *DHS* genes that relaxed the DHS negative feedback regulation [43,44]. The deregulation of DHS enzymes by the *sota* mutations boosted *in planta* AAA production by increasing carbon flux into the shikimate pathway. Notably, the enhanced AAA production was accompanied by the upregulation of carbon fixation probably to support high AAA production by supplying sufficient carbon sources through photosynthetic carbon metabolism [43]. Another genetic analysis of *Petunia* x *hybrida* transgenic lines revealed that the expression of *RPE1*, which encodes an E4P-producing d-ribulose-5-phosphate 3-epimerase enzyme in the pentose phosphate pathway, was somehow changed in response to the downstream Phe biosynthetic activity, regulating the supplied amount of carbon precursor from the pentose phosphate pathway into the shikimate pathway [45]. Although the mechanism underlying upregulated carbon assimilation remains unclear, these genetic results imply a tightly interconnected monitoring system between the upstream central carbon metabolism and the shikimate pathway to adjust the carbon allocation depending on carbon demand from the downstream AAA pathways.

In grasses (family Poaceae), the sensitivity of DHS enzymes to some metabolites has been changed to optimize the grass-specific production of AAA-derived compounds. Lignin is the most abundant aromatics, accounting for 30% of plant dry weight and is produced from Phe in most plant species [17,46]. Uniquely, grass species can biosynthesize a significant proportion of lignin from Tyr, but not only Phe (see the details in §9) [47–49]. To support high lignin production from Tyr, they use a grass-specific feedback-insensitive DHS isoform with organ-specific expression (e.g. internodes) to produce a high level of Tyr [50]. This grass feedback-resistant DHS enzyme is, to our knowledge, the first reported naturally occurring deregulating DHS enzyme. However, this deregulated grass DHS enzyme does not have sota-like mutations in its sequence, suggesting different mechanisms that change the DHS sensitivity to the downstream metabolites. Structural analysis of plant DHS enzymes will help identify key residue(s) for species-specific DHS feedback insensitivity and accelerate the development of plant DHS enzymes as a genetic tool to boost AAAs and aromatic natural products in plants.

Besides the metabolite-mediated negative feedback inhibition, plant DHS activity is associated with photosynthesis. Despite having similar *K*_m_ values of microbial and plant DHS enzymes for PEP, the *K*_m_ values for E4P in plant DHS enzymes are approximately 10 times higher than those of microbial DHS enzymes [40,42]. This discrepancy suggests that plant DHS enzymes have evolved to respond to fluctuation in E4P availability from the pentose phosphate pathways, including the Calvin–Benson cycle, which provides a significant amount of carbon precursor. Additionally, unlike microbial DHS enzymes, plant DHS activity necessitates reducing agents like 1,4-dithiothreitol, or thioredoxin *f*, which serves as a mediator of photosynthetic redox power in chloroplasts [42,51,52]. These photosynthesis-associated mechanisms probably play a regulatory role in maximizing DHS activity in the light (photosynthesis-active, carbon-sufficient condition) and downregulating it in the dark (photosynthesis-limited, carbon-deficient condition). In other words, the heterologous expression of functional plant-type DHS enzymes in non-photosynthetic microbes may be challenging owing to the lack of chloroplast-like carbon and redox availability in their cells. Also, we still have limited knowledge of how plants activate the DHS activity to produce AAAs in photosynthesis-limited tissues, such as roots.

## 4. Anthranilate synthase

Trp biosynthesis is initiated with the conversion of chorismate into anthranilate by AS. The AS complex forms a heterodimer or tetramer consisting of large α and small β subunits (ASα and ASβ, respectively) [53]. ASα binds to chorismate and is also allosterically inhibited by Trp to impair its conformational change that is necessary for chorismate conversion ([54,55]; figure 3). Arabidopsis *trp5−1* nucleotide substitution was identified as a feedback-insensitive ASα mutation that elevated the Trp level, suggesting that the AS reaction is critical to control carbon flux at the entry step of the Trp biosynthesis [56]. In most land plants, ASα enzymes are feedback-sensitive to Trp and encoded by at least two genes, one of which is inducibly expressed in response to development and/or biotic stresses likely to increase Trp-derived defence compounds [57–61]. Notably, *Ruta graveolens* possesses a Trp-feedback-resistant ASα isoform that probably contributes to the high production of lineage-specific Trp-derived alkaloids in the Rutaceae family, such as furoquinoline [59,62,63]. Also, a *Nicotiana tabacum* (tobacco) gene encoding feedback insensitive AS enzyme was cloned, while its physiological role in the metabolism of Trp-derived compounds in tobacco remains unclear [60]. Genes encoding these feedback-insensitive AS enzymes can be used for the metabolic engineering of Trp and Trp-derived natural products. Overexpression of the feedback-resistant ASα mutant isoform elevated the levels of Trp in rice calli, potato shoots, Arabidopsis and a forage legume *Astragalus sinicus* [61,64–67]. On the other hand, the accumulation of indole−3-ylmethyl glucosinolate, one of the major Trp-derived indole glucosinolates, was stimulated with little impact on the amount of camalexin, another Trp-derived defence compound, in the Arabidopsis transgenic line expressing the feedback-insensitive ASα mutant [66]. These metabolic engineering experiments propose feedback-insensitive ASα as a potential genetic tool to boost the accumulation of Trp, leading to the development of new crops with enhanced nutritional profiles or better resistance to resistance to pests and pathogens.

## 5. Chorismate mutase

Chorismate mutase (CM) converts chorismate into prephenate at the branching entry point of Phe/Tyr and Trp pathways. While microbes have bifunctional CM/prephenate dehydrogenase (PDH) enzymes, plant CM enzymes are monofunctional [68]. The CM activity is inhibited by Phe or Tyr and activated by Trp, allowing reciprocal flux regulation into either post-chorismate pathway ([69,70]; figure 3). Most flowering plants contain at least two CM isoforms, CM1 and CM2, that have distinct enzyme properties. In contrast to canonical feedback-sensitive CM1, the CM2 activity is not affected by either AAA, structural analysis of Arabidopsis CM1 enzyme identified mutations at a glycine residue and glycine/aspartate residues that abolish AAA allosteric regulation in CM2 and alter AAA specificity in CM1 and CM3, respectively [71]. Interestingly, all the CM enzymes isolated from a basal lineage of plant species, the moss/bryophyte *Physcomitrella patens*, the lycophyte *Selaginella moellendorffii*, and the basal angiosperm *Amborella trichopoda*, are activated by Trp with the limited effect of Tyr and Phe [72]. This evolutionary trajectory implies that plants have fine-tuned the CM sensitivity to AAAs at the branching point of post-chorismate pathways to balance the availability of each AAA for its derived specialized metabolites.

Another property of plant CM enzymes is their dual localization in plastids and cytosol. While many shikimate and AAA pathway enzymes, including AAA-sensitive CM isoforms, are localized in plastids, AAA-unregulated CM isoform is cytosolic owing to lacking of a transit peptide towards plastids at its N-terminus [70] (figure 2). The reverse genetic approach in Arabidopsis and petunia revealed that cytosolic CM isoform was responsible for cytosolic Phe production via phenylpyruvate that played significant roles in Phe-derived metabolism for wounding response and floral volatile emission [38,73].

## 6. Arogenate and prephenate dehydratases

Since lignin accounts for 15 to 32% of the total biomass in vascular plants, a sizeable amount of photosynthetically assimilated carbon is directed to the Phe biosynthesis pathway, which owns adaptable regulatory mechanisms in response to plant development and environmental adaption [4,74]. In Phe and Tyr biosynthesis, chorismate is commonly converted into prephenate by CM [68]. In most microbes, prephenate is dehydrated by prephenate dehydratase (PDT) to produce phenylpyruvate, which is further subjected to transamination to Phe by phenylpyruvate aminotransferases (PPY-AT) [13,41,75]. On the other hand, most plants display dominant carbon allocation to an alternative Phe pathway where PPA-AT produces arogenate, followed by dehydration of arogenate into Phe by ADT ([76,77]; figure 2). While monofunctional PDT enzymes exist in yeast, fungi, and bacteria [78,79], plants use some ADT-derived PDT enzymes that can use prephenate as an alternative substrate in Arabidopsis, petunia and conifer [73,80–85]. These PDT enzymes and one of the CM isoforms are localized to the cytosol, being proposed to be committed to the cytosolic Phe biosynthesis in plants (figure 2; [70,73,83,86]). While the cytosolic CM localization is mediated by the absence of functional transit peptide towards plastids at its C-terminus [70], the cytosolic PDT enzyme is created by transcription from an alternative transcription start site of a known gene encoding a plastidic ADT enzyme [73]. Phenylpyruvate is further converted into Phe by cytosolic PPY-AT that uses Tyr as an amino donor, indicating the interconnection between Tyr and Phe metabolism in the cytosol [83]. Compared with the well-studied microbial Phe production, the reason why plants have dual Phe biosynthetic pathways in plastids and the cytosol is still under debate owing to the limited impact of the cytosolic Phe pathway on total Phe production. Its potential roles include the rapid response of Phe production to abiotic and biotic stresses where plants need to produce Phe-derived specialized metabolites [73]. Also, phenylpyruvate, an intermediate in the cytosolic Phe pathway but not generated from the plastidic arogenate pathway, is used as an amino donor for Trp-derived auxin biosynthesis via Trp aminotransferases in petunia [21,87]. Given that phenylpyruvate is further catabolized for phenylpyruvate-derived specialized metabolites, including some aromatic volatiles [85,88], elevating cytosolic phenylpyruvate availability might be another possible benefit of the cytosolic Phe biosynthetic pathway to increase metabolic diversity of phenylpyruvate-derived aromatic natural products.

The PDT/ADT enzymes in microbes and plants are known to be feedback inhibited by Phe, the end-product of the pathways, to control the intercellular Phe level (figure 3). The enhancement of Phe production by introducing the artificially mutated feedback-insensitive ADT enzymes in Arabidopsis and rice highlights the important role of ADT allosteric regulation in maintaining the Phe level [89,90]. A recent phylobiochemical study discovered feedback-insensitive ADT enzymes that were widespread across vascular plants, with the ability to boost Phe production when overexpressed *in planta*. For example, *Nicotiana benthamiana* leaves transiently overexpressing deregulated ADT isoforms from *Physcomitrium patens* accumulated approximately 150-fold increased Phe levels compared to control leaves. The deregulation of ADT enzymes is mediated by several mutations onto the Phe allosteric binding domain of ADT enzymes, which is coincident with the emergence of vascular plants (tracheophytes) [91]. This indicates that the deregulation of ADT enzymes played a significant role in increasing precursor availability for Phe-derived compounds, primarily lignin, during the vascular plant evolution. Similarly, the cytosolic CM and PDT enzymes in petunia are also insensitive to Phe (figure 2) [70,73]. The deregulated Phe pathway in the cytosol probably contributes to rapid Phe production without the Phe-mediated pathway downregulation.

According to some classical reports published decades ago, the ADT enzyme activities from *Sorghum bicolor* and *Nicotiana silvestris* cell cultures were activated by Tyr possibly to redirect carbon flow from Tyr to Phe biosynthesis ([92,93]; figure 3). However, we do not have direct evidence that this Tyr-mediated ADT activation has metabolic impacts in plants.

## 7. Arogenate and prephenate dehydrogenases

Like Phe, Tyr is produced via two biosynthetic pathways in microbes and plants. Following the common biosynthetic route with Phe, prephenate is converted into 4-hydroxylphenylpyruvate (HPP) by PDH and subsequently transaminated reversibly by HPP aminotransferase (HPP-AT) to synthesize Tyr [94,95]. The generation of HPP leads to enzymatic Tyr degradation pathway where the degradation products are eventually catabolized in the mitochondrial tricarboxylic acid (TCA) cycle [96]. In the other route, arogenate, a transaminated product of prephenate by the PPA-AT enzyme, is oxidatively decarboxylated into Tyr by ADH, also known as TyrA. PDH and ADH enzymes are generally subjected to tight negative feedback inhibition by Tyr to adjust the Tyr level in cells (figure 3). With dominant PDH activity, microbes generally synthesize Tyr via the HPP route, whereas plants mainly use the arogenate pathway to produce Tyr in plastids [4,44,97]. Unlike ubiquitously detectable ADH activity across plant species, PDH activity has been detected only in the legume family [98,99]. A recent study identified genes encoding PDH enzymes in *Glycine max* (soybean) and *Medicago truncatula*. Inconsistent with canonical ADH enzymes, these legume PDH enzymes preferentially react with prephenate with limited residual activity for arogenate, are insensitive to Tyr, and are localized outside of plastids (figure 2; [99]). The structural analysis of soybean PDH determined Asn222 as a key residue that conferred PDH activity and Tyr insensitivity, and its conversion to aspartate was sufficient to change canonical Tyr-sensitive ADH enzymes to more relaxed PDH enzymes [100]. This finding indicates that amino acid alternation at this site was key to the emergence of legume-specific PDH enzymes [100]. Unfortunately, the physiological role of the cytosolic PDH-mediated Tyr biosynthesis in legumes remains largely unknown, as its Medicago knock-out mutant did not exhibit significant effects on biological phenotypes, including legume-specific nodulation, under standard growth conditions [101]. The only reported example of elevated Tyr production in legumes is that some Inga tree species accumulate a high amount of Tyr at up to 20% of dry weight to use Tyr and gallate conjugates as specialized defence metabolites [102]. Detailed phenotypic analyses of legume mutants defective in cytosolic PDH activity under stress conditions may provide new insight into its broader role in legume-specific biology.

Besides the Tyr insensitivity in legume PDH, convergently deregulated ADH enzymes have been identified in other plant groups. Caryophyllales is an order of angiosperm that includes beets, quinoa and spinach. Its unique chemical feature never seen in other plant orders is to lose the biosynthetic capability of anthocyanin, a red pigment derived from phenylalanine, but instead to be able to produce betalain, another red pigment synthesized from Tyr [15]. The lineage-specific betalain pigmentation is supported by high Tyr production mediated by the relaxation of ADT negative feedback inhibition. In *Beta vulgaris* (table beets), one of the two plastidic ADH enzymes (BvADHα), but not BvADHβ, exhibited relaxed sensitivity to Tyr and boosted Tyr production when heterologously expressed transiently in *Nicotiana benthamiana* and stably in Arabidopsis [44,103]. These findings demonstrate that the gene duplication of ancestral ADH isoforms and the following functional divergence and loss of Tyr sensitivity were key to the lineage-specific emergence of Tyr-derived pigment production during the Caryophyllales evolution [15]. Also, co-transformation of betalain biosynthetic genes with the *BvADHα* gene in tobacco and tomato further promoted heterologous betalain pigmentation by increasing Tyr precursor availability, highlighting its potential as a genetic tool to boost the production of Tyr-derived specialized metabolites in heterologous hosts [8,104].

As described in §3, another lineage-specific upregulation of Tyr biosynthesis contributes to the production of Tyr-derived lignin in grass species. In *Phyllostachys pubescens* (bamboo) shoots, where lignin production actively takes place, Tyr comprised approximately 60% of the total free amino acids, while the composition of Phe was only less than 1% of the total amino acids [105]. A recent ^13^CO_2_ feeding carbon flux analysis uncovered 10 times faster Tyr production in leaves and stems of *Brachypodium distachyon* and *Setaria viridis* than in those of Arabidopsis without a significant difference in Phe production capability [50]. In addition to the deregulated DHS enzyme, El-Azaz *et al*. [50] discovered grass-specific non-canonical ADH enzyme with low Tyr sensitivity that were highly expressed in growing stems. Like the deregulated beet ADH enzymes, the transient expression of deregulated Brachypodium ADH increased the Tyr level, and its combinational expression with deregulated Brachypodium DHS additively enhanced Tyr production. These findings highlight the biochemically fine-tuned regulation of the committed enzymes at the entry and exit steps of AAA biosynthesis that probably plays a crucial role in providing Tyr precursor for the Tyr-derived lignin pathway. However, phylogenetic evidence shows that the grass-deregulated ADH is not evolutionarily close to the Caryophyllene ADH or legume PDH, indicating that grass Tyr-insensitive ADH probably evolved independently in these three plant groups.

## 8. Phenylalanine hydroxylase

In human research, Phe degradation has been intensively studied because of its link to Phenylketonuria, a rare inherited metabolic disease in humans. In animals and microbes, Phe is directly hydroxylated into Tyr by phenylalanine hydroxylase (PAH) and eventually catabolized via the Tyr degradation pathway that leads to the TCA cycle in mitochondria [96,97,106]. In Phenylketonuria patients, inefficient or nonfunctional PAH results in the toxically elevated Phe level that inhibits the biosynthesis of AAA-derived neurotransmitters, such as serotonin and dopamine, triggering various metabolic dysfunctions, particularly in brains [107]. Therefore, PAH acts in a detoxification pathway to avoid excess Phe accumulation in cells. PAH enzymes belong to a family of tetrahydropterin-dependent aromatic amino acid hydroxylases (AAHs) that are widely spread in animals and microbes. Functional plastidic PAH enzymes are also encoded in the genomes of non-flowering photosynthetic eukaryotes, including green algae (*Chlamydomonas reinhardtii*), moss (*Physcomitrella patens*) and gymnosperm (*Pinus taeda*). The knock-out of the *PAH* gene in *Physcomitrella patens* elevated the levels of Phe and some Phe-derived phenylpropanoids, suggesting that the PAH enzyme redirects carbon flux from Phe to Tyr ([108]; figure 3). By contrast, orthologous *AAH* genes are lacking in angiosperm genomes, which have made the plant Phe degradation pathway obscure for a long time. The absence of the animal-type Phe degradation pathway might be compensated for by large carbon flux into Phe-derived phenylpropanoids, especially for lignin biosynthesis, during the angiosperm evolution [17,18,46]. As land plants have evolved to increase Phe precursor availability for lignin biosynthesis (e.g. deregulation of ADT enzymes and cytosolic Phe pathway, §6), the loss of the Phe-to-Tyr conversion sounds reasonable to avoid wasting Phe precursor for lignin production. An alternative explanation is the emergence of new hydroxylases that have convergently evolved to convert Phe into Tyr. Indeed, plant PAH activity was only reported from *Spinacia oleracea* (spinach) leaves six decades ago [109]. However, no significant follow-up reports have been published since then. Consistently, no PAH activity was detected from Tyr hydroxylase activity of *Mucuna pruriens*, a species in legume, which mediates a similar reaction that forms 4-dihydroxyphenylalanine (L-DOPA) from Tyr [110]. Therefore, it is still under debate if flowering plants possess the animal-type Phe degradation pathway.

## 9. Phenylalanine and phenylalanine/tyrosine ammonia-lyase

Phenylalanine ammonia-lyase (PAL) enzymes catalyse the first and committed reaction of Phe deamination into cinnamate, initiating phenylpropanoid biosynthesis (figure 3). The PAL reaction plays a critical role in determining the carbon flux into the phenylpropanoid pathway. Consistently, the overexpression of the plant *PAL* gene leads to upregulation of phenylpropanoid production [111]. Given the bottleneck of carbon flux from Phe into the entire phenylpropanoid pathway, plant PAL activity is subjected to complex multilayered regulatory mechanisms at both transcriptional and post-transcriptional levels [112–114]. The expression of *PAL* genes is inducible to various stresses, such as high light, wounding and pathogen attacks, to cope with such stresses by promoting the production of defensive phenylpropanoid compounds, such as anthocyanin and coumarin [20,115,116]. At the post-transcriptional level, the PAL ubiquitination occurs with kelch motif-containing F-box proteins to facilitate the PAL degradation and turnover [117–119]. Additionally, PAL enzymes are negatively regulated by pathway intermediates, including cinnamate, a product of PAL enzymes [120–126]. Collectively, plant PAL activity is complexly regulated to act as a gateway to phenylpropanoid biosynthesis and the following lignin production from Phe.

In addition to the well-established biosynthesis of Phe-derived lignin, grass species can produce lignin from Tyr via an alternative biosynthetic route. It was reported more than half a century ago that radioactive carbon (^14^C) derived from Tyr was incorporated into grass lignin, proposing the presence of tyrosine ammonia-lyase (TAL) activity that produces *p*-coumarate directly from Tyr without generating cinnamate (figure 3; [47]). Barros *et al.* [48] identified, out of eight *PAL* genes in the model grass Brachypodium, a gene encoding bifunctional phenylalanine/tyrosine ammonia-lyase (PTAL) that catalyses Tyr into *p*-coumarate with even higher *K*_m_ value towards Phe than the other seven monofunctional PAL enzymes. Brachypodium RNAi knock-down and stable carbon isotope (^13^C) feeding experiments revealed that the PTAL-mediated Tyr-derived lignin pathway accounted for approximately half of the total lignin. This result demonstrates that, with increased Tyr precursor availability by grass-specific feedback-insensitive DHS and ADH, grass species probably evolved to use the dual lignin biosynthetic pathways derived from Tyr and Phe. However, we still have limited knowledge of why grass plants need this second pathway. Since the syringyl (S)-rich lignin and cell wall-bound coumarates were preferentially produced in the *PTAL* knock-down line, the PTAL enzyme may contribute to the grass-specific lignin composition. Alternatively, the PTAL pathway can produce *p*-coumarate without generating cinnamate, the product of PAL enzymes that can feedback inhibit the PAL enzymatic activity. Given that Brachypodium PAL and PTAL enzymes showed comparable sensitivity to cinnamate, the PTAL route can escape from the cinnamate-mediated feedback inhibition, maintaining the high carbon flux into the phenylpropanoid pathway [48,114]. Besides the feedback regulation, understanding how the PTAL enzyme is regulated differentially from the PAL enzymes may provide a cue into the role of PTAL in grass-specific phenylpropanoid metabolism. Also, the genetic basis underlying the switching mechanism that determines the PAL and TAL activities is another interesting topic to address how Tyr-derived lignin pathway has emerged together with the high Tyr production during grass evolution.

## 10. Trends in the diversification of aromatic amino acid metabolic enzymes

**Figure 4.**
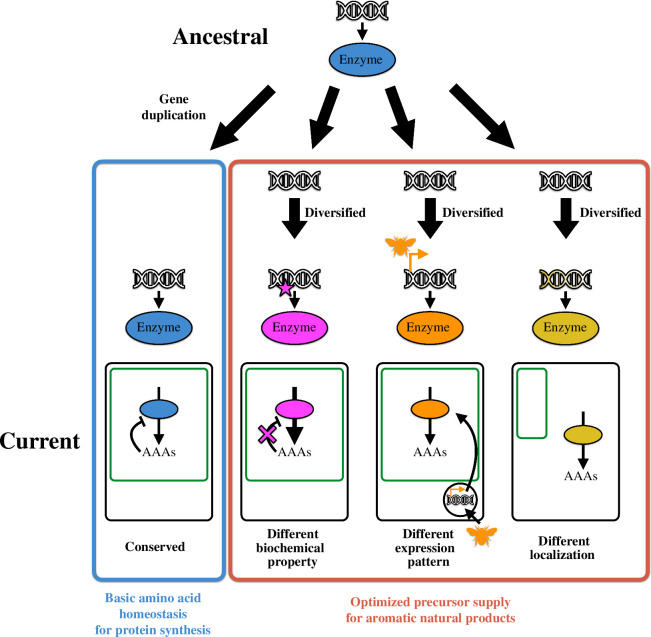
Trends in the diversification of aromatic amino acid metabolic enzymes. A single copy of an ancestral AAA metabolism gene is duplicated into multiple isoforms. Some of them are subsequently diversified to change their biochemical property, expression pattern and/or subcellular localization (magenta, orange and yellow, respectively). These divergent AAA metabolic enzymes probably play a critical role in optimized precursor supply for the biosynthesis of aromatic natural products. On the other hand, the isoform that is kept conserved to maintain the original functions (blue) probably contributes to basic amino acid homeostasis for protein synthesis.

Unlike the highly divergent specialized metabolism, primary metabolism, such as amino acid biosynthesis and catabolism, is generally assumed to be conserved among the kingdoms. In this review, I showcased the diversification of AAA metabolism with a focus on several metabolic enzymes that catalyse critical steps in AAA metabolism. In most cases, we can see trends in the diversification of the enzymes involved in AAA biosynthesis and catabolism. Like microbes, ancestral plants might have a single isoform of each enzyme in AAA metabolism that was subsequently duplicated into two or multiple copies of the enzyme. After the gene duplication, some isoforms have evolved to increase (or optimize) precursor availability of certain AAA-derived aromatic natural products that are critical for the kingdom- or lineage-specific development or adaptation (figure 4; [1,127,128]). In this evolutionary path, gene duplication is key to the evolution of AAA metabolism, which is consistent with the diversification of specialized metabolism [2,129–131]. The most notable lesson specific to plant primary metabolism is that, whenever plants have diversified AAA pathways, the original AAA pathway/enzyme has been conserved to maintain the basic capability of amino acid homeostasis, which probably plays an important role in general biological functions, such as protein synthesis. In other words, no plant species have been reported so far that lose the canonical AAA pathway or enzymes. The importance of basic AAA homeostasis is supported by some publications which reported that the artificial imbalance of AAA metabolism negatively impacted various house-keeping functions, such as plant growth [37,42,44]. These facts indicate plants’ diversification strategy where AAA metabolism has evolved divergently without affecting the basic amino acid homeostasis presumably for protein synthesis. Stated differently, plants can distinguish the conserved and divergent AAA metabolic pathways for protein synthesis and aromatic natural products, respectively, through an unknown mechanism, which will be the next question to be addressed. Another trend is that AAA metabolism has never been diversified unless plants evolved to produce significant amounts of AAA-derived aromatic natural products in a kingdom/lineage-specific manner. It is predicted that the emergence of a new AAA-derived specialized metabolic pathway might put evolutionary pressure on AAA metabolism to force it to provide more precursors for the downstream specialized metabolism. This highlights that the evolution of AAA metabolism has played a key role as a driving force in expanding the chemical diversity of plant aromatic natural products. Also, this implication may answer a long-standing question in biology: ‘why have animals not evolved the shikimate and AAA biosynthetic pathways like plants?’ Although plants and animals consume a large portion of cellular resources for multicellular development, plants also have to spend these carbon and energy resources to support the energy-intensive AAA biosynthetic pathway for the production of aromatic natural products that have allowed them to survive under challenging environmental conditions on land [132]. Animals, on the other hand, do not need to produce such specialized aromatic metabolites, which is probably one of the key factors that allows animals to forgo the development of energy-intensive AAA biosynthetic pathways.

The diversification of AAA metabolism-related enzymes can be classified into three categories: (i) biochemistry, (ii) transcription, and (iii) localization (figure 4). The biochemical diversification accompanies modified protein characteristics from those of the original enzymes and can be further divided into three subtypes. First, many of the committed enzymes in AAA biosynthesis are subjected to negative feedback inhibition mediated by the pathway end-products (and sometimes intermediates) to control the carbon flux into the pathways. The emergence of feedback-resistant isoforms upregulates their enzymatic activity, increasing carbon flux into the pathway. Second, changing substrate specificity redirects or bypasses carbon allocation in the pathway. Third, redox-regulated enzymes enable efficient carbon allocation coupled with photosynthesis. Another similar case of biochemical diversification in plant primary metabolism is isopropylmalate synthase (IPMS), an enzyme in leucine biosynthesis that is feedback inhibited by leucine [133]. In *Solanum lycopersicum* (cultivated tomatoes), one of the IPMS isoforms is a leucine-resistant enzyme that increases carbon flux into the leucine biosynthesis, contributing to high production of leucine-derived acylsugars in tomato trichomes [134]. In many cases of biochemical diversification in AAA metabolism, naturally occurring genetic mutations that change the biochemical properties of the enzymes have been identified, offering exciting opportunities to enhance endogenous metabolic pathways of AAA-derived specialized compounds and even to reconstruct plant natural product pathways in heterologous systems through synthetic biology [8]. The dominant nature of most of these genetic mutations is also an advantage when heterologously expressed. The recent breakthrough in base editing technology has made it easier to introduce genetic mutations into various plant species, accelerating the use of these genetic mutations as a natural metabolic booster for plant metabolic engineering.

Transcriptional divergence in AAA metabolic genes offers an efficient precursor supply that enables flexible production of AAA-derived natural products. Plants often inducibly produce AAA-derived metabolites in a tissue-dependent manner or response to environmental stresses, such as lignin in growing stems and defence compounds against pathogen attack. If the production of AAA-derived metabolites is not spontaneously enhanced with the increased supply of AAA precursor, the precursor shortage will limit the downstream biosynthetic efficiency of aromatic natural products. By contrast, constitutively activated AAA production would waste carbon and energy required for AAA biosynthesis, as excessively accumulated amino acids are transported into vacuoles [135], where cytosolic AAA catabolic enzymes cannot access. Given that AAAs are the most energy-intensive among the 20 proteogenic amino acids to synthesize in plant cells [132], co-expression of biosynthetic gene sets for AAAs and AAA-derived natural products are the most efficient and leanest way to synthesize aromatic natural products. An excellent example of this is the co-expression of *ADH* and *PTAL* genes together with other lignin biosynthetic genes in growing grass stem internode tissues where Tyr-derived lignin production actively takes place [50]. In Arabidopsis, a MYB transcription factor HIG1/MYB51 regulating Trp-derived indole glucosinolate biosynthesis activates a gene encoding Trp-insensitive DHS1 enzyme to elevate the Trp precursor amount for indole glucosinolate defence compounds in response to biotic stresses [136]. Combinational gene expression of deregulated AAA metabolic enzymes with aromatic specialized metabolic genes is one of the strategies for efficient chemical production plants have developed during the evolution but also can be used for us to conduct large-scale plant metabolic engineering of aromatic natural products [8].

We have had limited knowledge about the subcellular location of the shikimate and AAA biosynthetic pathway until recently. AAAs are dominantly synthesized in plastids (chloroplasts), probably because of the large amounts of carbon and energy sources available from photosynthesis. This aspect is significantly critical, as plants need a lot of carbon and energy (ATP) to build chemically stable aromatic rings in AAAs [132]. The cytosolic AAA biosynthetic pathway has been proposed, along with a growing body of evidence that several AAA biosynthetic enzymes are located in the cytosol. Possible roles of the cytosolic AAA pathway in plants include providing AAA precursors to the cytosolic downstream pathways more efficiently, to generate pathway intermediates (e.g. phenylpyruvate) that cannot be synthesized from the plastidic pathway, to act as a backup pathway in immature forms of plastids with limited availability of carbon and energy resources, or in case the plastidic dominant pathway is somehow downregulated (e.g. defects in chloroplast development) and to spatially escape from tight regulation or potential damages in plastids (e.g. plastidic proteases) [38]. More detailed genetic, cell biology, and carbon flux analysis in various plant species will be needed to understand how the cytosolic AAA pathway is ubiquitously distributed among the plant kingdom, is regulated at a post-transcriptional level, and contributes to the metabolism of AAAs and AAA-derived natural compounds.

## 11. Perspectives

The recent phylogenomic, biochemical and metabolomic studies have uncovered the diversification of plant AAA metabolism that is associated with lineage-specific production of AAA-derived natural products. Remarkably increasing genomic and transcriptional resources and recently developed genome editing technology are accelerating the discovery and use of such natural metabolic innovations for plant-based chemical engineering [131,137–140]. However, we perhaps know only the limited number of cases in the diversification of plant AAA metabolism and aromatic natural product biosynthesis. It is estimated that approximately 400 000 extant species of vascular plants on the Earth produce between 200 000 and 1 million metabolites [3,141–143]. Our limited knowledge of plant chemical diversity may be a bottleneck in harnessing evolutionary diversification of primary metabolism, including AAA biosynthesis and catabolism, for future plant metabolic engineering in the next decades. Indeed, public metabolomic resources of various plant species where we can deposit metabolomic phenotypes with taxonomic information are even less available than those of genomics or transcriptomics. With advanced mass spectrometry-based metabolomics techniques, analysing the chemical composition of various tissues of as many plant species as possible, obtaining metabolite data containing raw chromatography and absolute quantification of several major primary metabolites, and sharing them with genomic and taxonomical information to the research community may give us clues into re-discovery of plant chemical diversity we have missed for a long time [144]. This knowledge could lead to crops that are better able to withstand environmental stress or to improve production of chemicals, biomaterials and other plant-based products.

## Data Availability

This article has no additional data.
